# Cadonilimab (PD-1/CTLA-4 bispecific antibody) combination therapy for driver gene-negative advanced NSCLC: a single-center retrospective real-world study

**DOI:** 10.3389/fonc.2026.1718151

**Published:** 2026-02-16

**Authors:** Lulin Zeng, Yan Xiang, Ao Sun, Kaihua Lu

**Affiliations:** Department of Oncology, The First Affiliated Hospital of Nanjing Medical University, Nanjing, China

**Keywords:** bispecific antibody, Cadonilimab, driver gene-negative, immunotherapy, non-small cell lung cancer

## Abstract

**Objective:**

Immunotherapy has made significant progress in the treatment of advanced non-small cell lung cancer (NSCLC). However, treatment options for driver gene-negative patients remain limited. This study evaluated the efficacy and safety of Cadonilimab (AK104), a bispecific PD-1/CTLA-4 antibody, in this population.

**Methods:**

We retrospectively analyzed real-world data from driver gene–negative advanced NSCLC patients treated with AK104. Outcomes included objective response rate (ORR), disease control rate (DCR), and progression-free survival (PFS).

**Results:**

Among 30 patients, AK104 combination therapy achieved an ORR of 23.3%, DCR of 80%, and median PFS (mPFS) of 6.3 months. The combination of AK104 with chemotherapy and other anti-angiogenic inhibitors (AAI) achieved a notable mPFS of 11.1 months. First-line treatment (n=12) yielded ORR 50%, DCR 100%, and mPFS 13.3 months; second-line (n=6) ORR 16.7%, DCR 100%, mPFS 7.7 months; beyond second-line (n=12) ORR 0%, DCR 50%, mPFS 2.6 months. No significant difference in mPFS was observed between PD-L1–positive and PD-L1–negative patients (4.5 vs. 3.0 months, *P* = 0.76). Common adverse events included anemia (66.7%), leukopenia (63.3%), neutropenia (56.7%), and thrombocytopenia (53.3%), with grade 3 events in 16.7%. One patient discontinued due to immune-related pancreatitis, and no deaths occurred.

**Conclusions:**

This study confirms the promising efficacy and acceptable safety profile of AK104 combination therapy in patients with driver gene-negative advanced NSCLC. These findings collectively support the need for further large-scale prospective studies to validate its clinical utility.

## 1 Introduction

Lung cancer is one of the most common malignancies, with non-small cell lung cancer (NSCLC) accounting for approximately 85% of cases. About 70% of newly diagnosed patients have advanced disease, and fewer than 50% harbor driver gene mutations (1, 2). In recent years, immune checkpoint inhibitors targeting the programmed cell death protein 1 (PD-1) and its ligand programmed cell death ligand 1 (PD-L1) have made significant progress in clinical research. Extensive clinical evidence has demonstrated that PD-1/PD-L1 inhibitors provide significant efficacy and manageable safety profiles in both monotherapy and combination settings. To date, PD-1/PD-L1 inhibitor therapy with or without chemotherapy has become the standard treatment for advanced NSCLC patients with driver gene-negative status (3). However, significant heterogeneity persists in current treatment approaches, with substantial clinical benefit observed in patients exhibiting high PD-L1 expression (tumor proportion score [TPS] ≥50%) and limited clinical response in those with PD-L1–negative tumors (TPS <1%) (4–7). More critically, most patients receiving immunotherapy ultimately develop primary or acquired resistance (8). These observations emphasize the need to develop effective antitumor therapeutic strategies for patients with advanced NSCLC lacking driver gene mutations.

With the rapid development of immune checkpoint inhibitors, the therapeutic landscape for patients with driver gene-negative advanced NSCLC has evolved substantially in recent years. Cadolinimab (AK104), a bispecific antibody targeting PD-1 and CTLA-4, has demonstrated superior clinical efficacy in multiple solid tumor clinical trials by synergistically inhibiting both immune checkpoint pathways (9, 10). This agent has received approval from China’s National Medical Products Administration (NMPA) for two indications: first-line treatment of locally advanced unresectable or metastatic gastric/gastroesophageal junction adenocarcinoma in combination with fluoropyrimidine and platinum-based chemotherapy, and treatment of recurrent or metastatic cervical cancer in patients who have previously failed platinum-based chemotherapy. In the NSCLC setting, a Phase Ib study (11) reported that AK104 combined with anlotinib achieved the objective response rate (ORR) of 60% and the disease control rate (DCR) of 95% in first-line treatment of advanced NSCLC. Data from a Phase II study (12) indicated that AK104 monotherapy as a later-line treatment for advanced NSCLC patients achieved a best DCR of 40% and a median progression-free survival (mPFS) of 1.91 months. Although these findings provide preliminary evidence supporting the therapeutic potential of AK104 in advanced NSCLC, real-world data evaluating the efficacy and safety of AK104-based combination regimens in the driver gene–negative NSCLC population remain limited.

Therefore, the present study was conducted to further assess the clinical efficacy and safety of AK104-based combination therapy in patients with advanced non-small cell lung cancer (NSCLC) lacking driver gene mutations, using a single-center patient cohort. This analysis aims to provide more precise guidance for the management of this patient population and to inform the optimization of therapeutic regimens in clinical practice.

## 2 Materials and methods

### 2.1 Study design and patient population

This study collected clinical data from 30 patients with advanced NSCLC who received AK104 combination therapy at the First Affiliated Hospital of Nanjing Medical University between September 2022 and October 2024. Data collection was finished on December 31, 2024. The clinical data collected encompassed demographic characteristics (including sex and age), smoking history, ECOG performance status, tumor stage, histological subtype, biomarker profiles such as PD-L1 expression, and treatment history, including detailed documentation of administered regimens and the number of treatment lines.

The inclusion criteria were as follows: patients aged 18 years or older; histopathologically confirmed unresectable locally advanced or metastatic NSCLC; absence of driver gene alterations, including *EGFR*, *ALK*, *ROS1*, *MET*, *HER2*, *BRAF*, *NTRK*, and *RET*; at least one measurable lesion according to RECIST 1.1 criteria; and complete clinical records, including at least two documented treatment regimens and one or more efficacy assessments performed at this institution.

All patients received AK104-based combination therapy at the discretion of their treating physicians as part of routine clinical management. At the time of treatment, AK104 was not approved in China for the treatment of NSCLC and was not administered within any interventional clinical trial. The decision to use AK104 was made on an individual basis after multidisciplinary discussion. The study was conducted in strict accordance with the Declaration of Helsinki and was approved by the Ethics Committee of the First Affiliated Hospital of Nanjing Medical University (Ethics Approval No. 2023-SR-847). All enrolled patients provided written informed consent.

### 2.2 Efficacy assessment of treatment

This study employed a systematic review and analysis methodology to evaluate the clinical characteristics, efficacy, and safety of the AK104 combination therapy regimen. The primary endpoints included PFS, ORR, and DCR. Tumor response was strictly assessed according to the Response Evaluation Criteria in Solid Tumors (RECIST 1.1), categorizing lesion changes as complete response (CR), partial response (PR), stable disease (SD), or progressive disease (PD). PFS was defined as the interval from initiating AK104 combination therapy to radiologically confirmed disease progression or death. ORR was defined as the proportion of patients achieving CR or PR. DCR represented the cumulative proportion of CR, PR, or SD cases. Safety was evaluated according to the Common Terminology Criteria for Adverse Events (CTCAE) version 5.0, considering both the overall incidence of adverse events (AEs) and the incidence of grade ≥3 AEs.

### 2.3 Statistical analysis

Data analysis was conducted using SPSS 26.0 and R software (version 4.3.2). Quantitative variables, including clinical characteristics and demographic data, were summarized with medians and ranges, while categorical variables were presented as frequencies and percentages. PFS was calculated using the Kaplan-Meier method, with survival curves plotted. The log-rank test was applied to evaluate differences in PFS. P < 0.05 was considered statistically significant.

## 3 Results

### 3.1 Patient characteristics

A total of 30 patients were included in this study, and their baseline characteristics are summarized in **Table 1**. Most patients (83.3%, 25/30) were classified as stage IV. Histopathological subtypes included non-squamous NSCLC (60%, 18/30) and squamous NSCLC (40%, 12/30). Treatment regimens consisted of AK104 combined with chemotherapy in 5 patients, AK104 combined with anlotinib in 4 patients, AK104 combined with chemotherapy and anlotinib in 12 patients, and AK104 combined with chemotherapy plus other anti-angiogenic inhibitors(AAI) such as bevacizumab or endostatin in 9 patients. Chemotherapy regimens involved platinum-based doublets, combining platinum agents with taxanes, gemcitabine, or pemetrexed, as well as single-agent maintenance therapy.

**Table 1:** Patients baseline characteristics and treatment therapies.

Characteristics	N=30(%)
Age, median (years)	67.5(48-80)
Gender	
Male	27(90.0)
Female	3(10.0)
Smoking history	
Never	9(30.0)
Former	21(70.0)
Histological type	
Non-squamous	18(60.0)
Squamous	12(40.0)
Stage	
IIIB	5(16.7)
IV	25(83.3)
ECOG-PS score	
0	11(36.7)
1	19(63.3)
Brain metastases	
Yes	5(16.7)
No	25(83.3)
Liver metastases	
Yes	2(6.7)
No	28(93.3)
Bone metastases	
Yes	11(36.7)
No	19(63.3)
Organs with metastases	
0	5(16.7)
1	10(33.3)
2	7(23.3)
3	3(10.0)
≥4	5(16.7)
PD-L1 expression	
Negative	9(30.0)
Positive	8(26.7)
Unknown	13(43.3)
Prior anti-PD-1 therapy	
Yes	17(56.7)
No	13(43.3)
AK104 treatment line	
1L	12(40.0)
2L	6(20.0)
	12(40.0)
AK104 therapeutic schemes	
AK104+chemotherapy	5(16.7)
AK104+Anlotinib	4(13.3)
AK104+chemotherapy+other AAI	9(30.0)
AK104+chemotherapy+Anlotinib	12(40.0)

ECOG, Eastern Cooperative Oncology Group; PS, performance status; PD-L1, programmed cell death ligand 1; PD-1, programmed cell death-1; 1L, first line; 2L, second line; AAI, anti-angiogenesis inhibitor (including bevacizumab and endostatin).

Notably, 12 patients (40%) received first-line therapy, including 2 patients (16.7%) treated in combination with chemotherapy, 1 patient (8.3%) treated in combination with anlotinib, 2 patients (16.7%) treated in combination with chemotherapy plus anlotinib, and 7 patients (58.3%) treated in combination with chemotherapy plus other anti-angiogenic inhibitors(AAI). In the second-line setting, 6 patients (20%) received second-line therapy, including 1 patient (16.7%) treated in combination with chemotherapy, 1 patient (16.7%) treated in combination with anlotinib, 3 patients (50%) treated in combination with chemotherapy plus anlotinib, and 1 patient (16.7%) treated in combination with chemotherapy plus other AAI. And 12 patients (40%) received third-line or subsequent treatment, including 2 patients (16.7%) treated in combination with chemotherapy, 2 patients (16.7%) treated in combination with anlotinib, 7 patients (58.3%) treated in combination with chemotherapy plus anlotinib, and 1 patient (8.3%) treated in combination with chemotherapy plus other AAI. Furthermore, all 18 patients (60%) treated in the second-line or later settings had prior exposure to platinum-based chemotherapy. PD-L1 expression was assessed using the 22C3 antibody platform (Dako PD-L1 IHC 22C3 pharmDx). A total of 17 patients (56.7%) completed testing, of whom 52.9% (9/17) were PD-L1-negative (TPS < 1%) and 47.1% (8/17) were PD-L1-positive (TPS ≥ 1%). Regarding distant metastases, 16.7% of patients had brain metastases, 36.7% had bone metastases, and 16.7% had multi-organ metastases.

### 3.2 Efficacy

**Figure 1:**
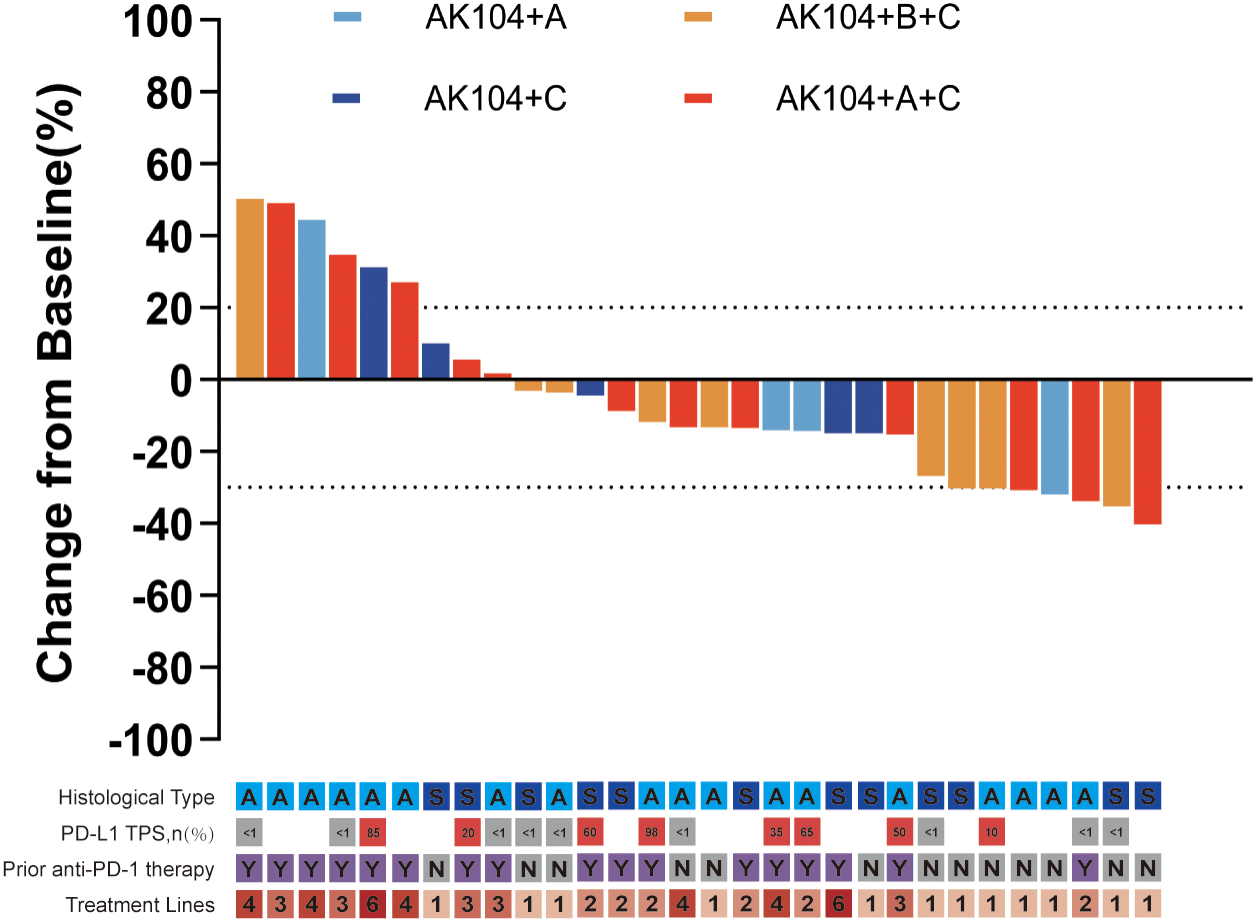
Change from baseline in target lesion size for each patient. PD, progressive disease; SD, stable disease; PR, partial response. In the legend: A, non-squamous; S, squamous; PD-L1 TPS, programmed cell death ligand-1 (tumor proportion score); Y, yes; N, no. AK104+C, AK104 combined with chemotherapy; AK104+A, AK104 combined with anlotinib; AK104+A+C, AK104 combined with anlotinib and chemotherapy; AK104+B+C, AK104 combined with other anti-angiogenesis inhibitors (including bevacizumab and endostatin) and chemotherapy.

**Figure 2:**
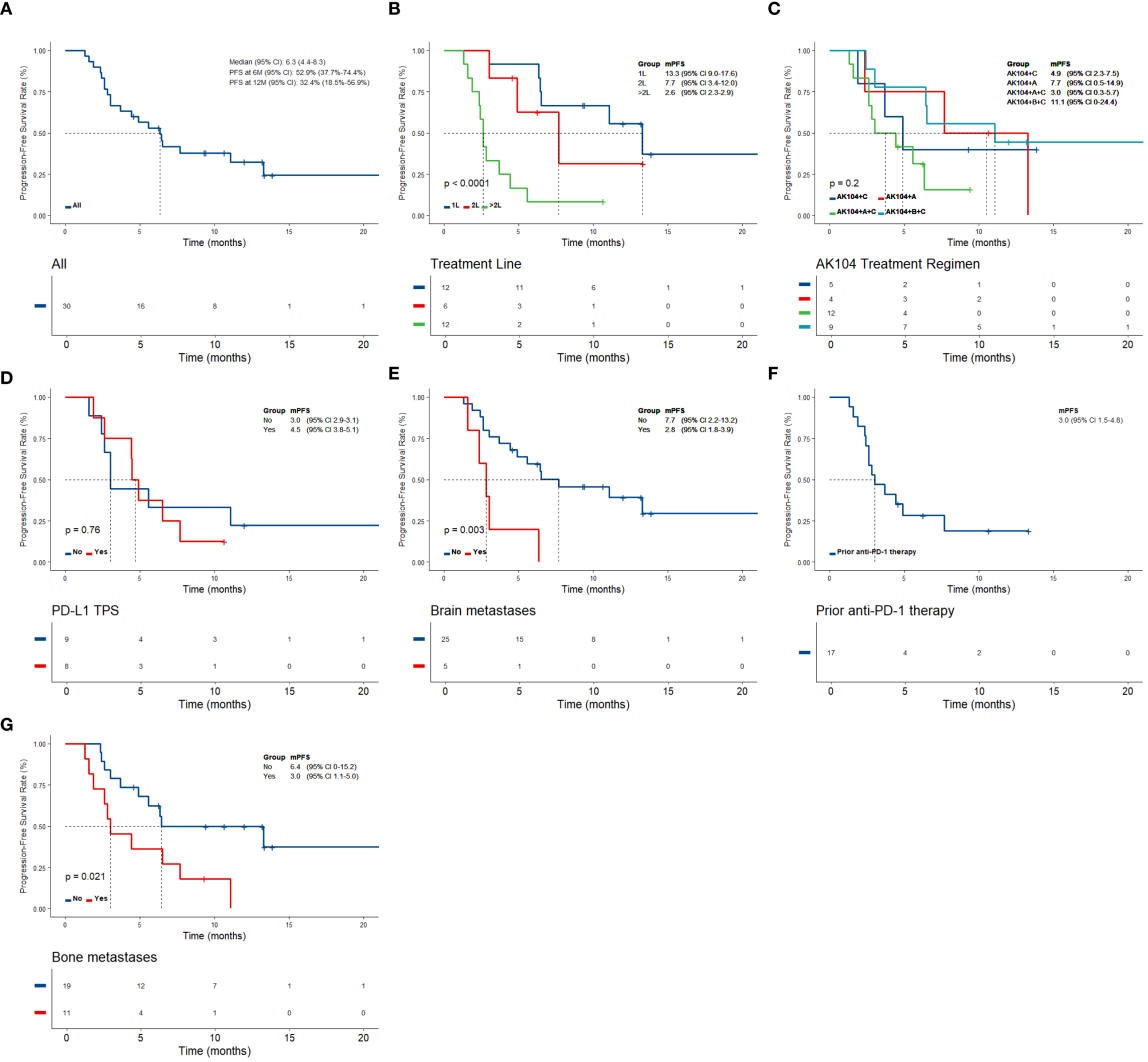
Kaplan–Meier estimates of PFS according to **(A)** the overall NSCLC population, **(B)** AK104 treatment line, **(C)** AK104 treatment regimen, **(D)** PD-L1 expression, **(E)** brain metastases, **(F)** prior anti-PD-1 therapy, **(G)** bone metastases. (PFS at 6M, 6-month PFS rate; PFS at 12M, 12-month PFS rate; AK104+C, AK104 combined with chemotherapy; AK104+A, AK104 combined with anlotinib; AK104+A+C, AK104 combined with anlotinib and chemotherapy; AK104+B+C, AK104 combined with other anti-angiogenesis inhibitors (including bevacizumab and endostatin) and chemotherapy.

As of the December 2024 follow-up, among the 30 patients with stage IIIB/IV NSCLC, 23.3% (7/30) achieved PR, 56.7% (17/30) showed SD, and 20% (6/30) experienced PD. The ORR was 23.3% (7/30), and the DCR was 80% (24/30) (**Table 2**). Changes in target lesion size are illustrated in **Figure 1**. Survival analysis revealed the mPFS of 6.3 months (95% CI, 4.4–8.3 months). The 6-month PFS rate was 52.9% (95% CI, 37.7%-74.4%), and the 12-month PFS rate was 32.4% (95% CI, 18.5%-56.9%) (**Figure 2A**).

**Table 2:** Tumor response to AK104 in the overall population and the subgroup population.

Response category	Overall (N = 30)	Site of metastases			PD-L1 TPS(%)		Prior anti-PD-1 therapy		AK104 treatment line			AK104 treatment combined schemes			
		Brain (N = 5)	Liver (N = 2)	Bone (N = 11)	Negative (N = 9)	Positive (N = 8)	Yes (N = 17)	No (N = 1)	1L (N = 12)	2L (N = 6)	>2L (N = 12)	Chemo (N = 5)	Alo (N = 4)	Chemo+ Alo (N = 12)	Chemo+ Other AAI (N = 9)
ORR,n(%) 95%CI	7(23.3) [10.6-42.7]	2(40.0) [11.8-76.9]	0 [0-65.8]	1(9.1) [1.6-37.7]	2(22.2) [6.3-54.7]	1(12.5) [2.2-47.1]	1(5.9) [1.0-27.0]	0 [0-79.3]	6(50) [25.4-74.6]	1(16.7) [3.0-56.4]	0 [0-24.2]	0 [0-43.4]	1(25.0) [4.6-69.9]	3(25.0) [8.9-53.2]	3(33.3) [12.1-64.6]
CR	0	0	0	0	0	0	0	0	0	0	0	0	0	0	0
PR	7(23.3)	2(40.0)	0	1(9.1)	2(22.2)	1(12.5)	1(5.9)	0	6(50.0)	1(16.7)	0	0	1(25.0)	3(25.0)	3(33.3)
SD	17(56.7)	0	1(50.0)	6(54.5)	5(55.6)	6(75)	10(58.8)	1(100)	6(50.0)	5(83.3)	6(50.0)	4(80.0)	2(50.0)	6(50.0)	5(55.6)
PD	6(20.0)	3(60.0)	1(50.0)	4(36.4)	2(22.2)	1(12.5)	6(35.3)	0	0	0	6(50.0)	1(20.0)	1(25.0)	3(25.0)	1(11.1)
DCR,n(%) 95%CI	24(80.0) [60.9-91.6]	2(40.0) [11.8-76.9]	1(50.0) [9.5-90.5]	7(63.6) [35.4-84.8]	7(77.8) [45.3-93.7]	7(87.5) [52.9-97.8]	11(64.7) [41.3-82.7]	1(100) [20.7-100]	12(100) [75.6-100]	6(100) [60.9-100]	6(50.0) [25.4-74.6]	4(80.0) [37.6-96.4]	3(75.0) [30.1-95.4]	9(75.0) [46.8-91.1]	8(88.9) [56.5-98.0]

CR, complete response; PR, partial response; SD, stable disease; PD, progressive disease; ORR, objective response rate; DCR, disease control rate; PD-L1 TPS, programmed cell death ligand-1 (tumor proportion score); Negative, TPS<1%; Positive, TPS≥1%; 1L, first line; 2L, second line; Alo, Anlotinib; AK104, Cadonilimab; AAI, anti-angiogenesis inhibitor (including bevacizumab and endostatin).

**Figure 3:**
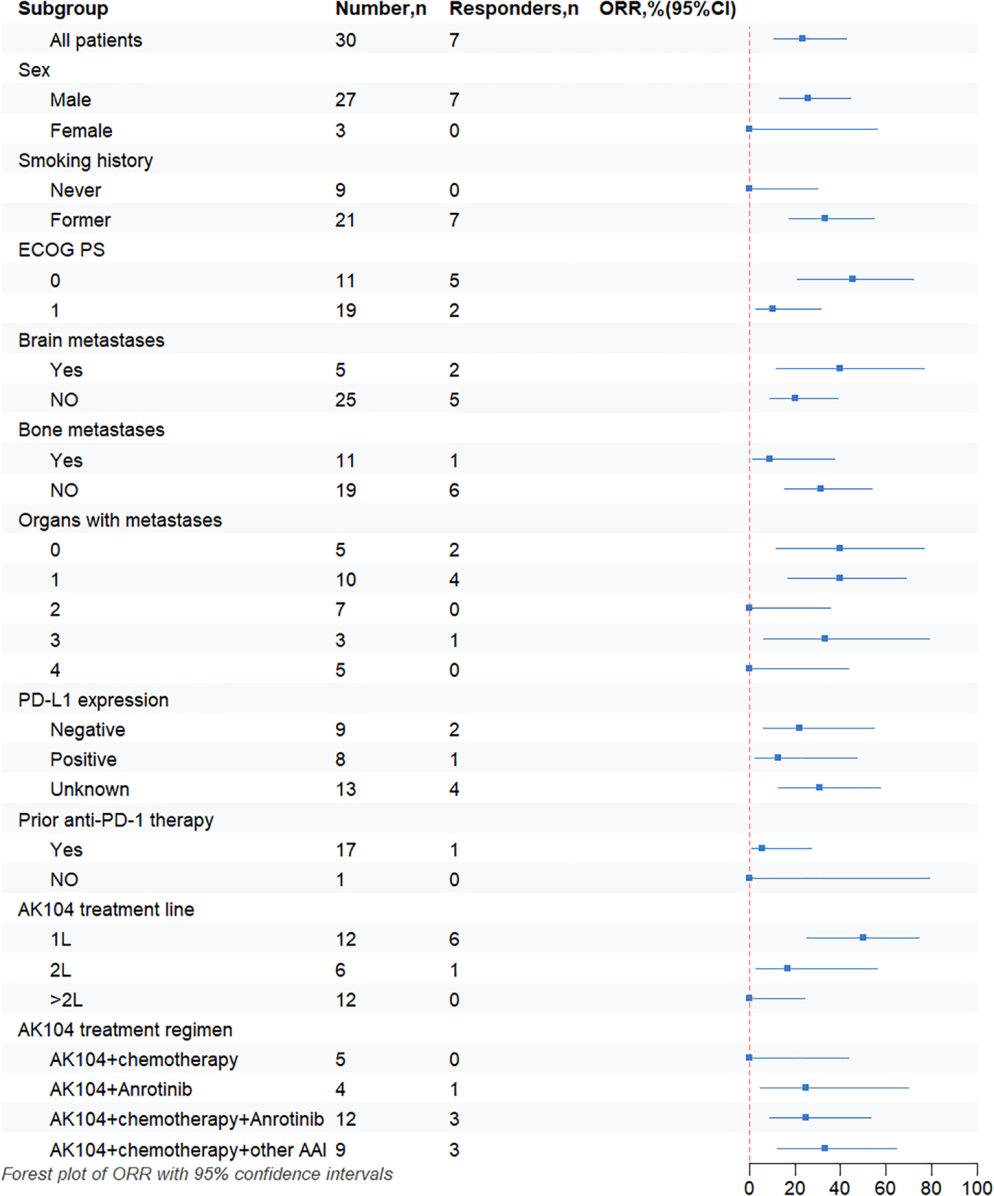
Shows a forest plot of subgroup analysis of objective response rates by baseline demographic and disease characteristics. ORR, objective response rate; ECOG, Eastern Cooperative Oncology Group; PS, performance status; 1L, first line; 2L, second line; PD-1, programmed cell death-1; PD-L1, programmed cell death-ligand 1; CI, confidence interval; AAI, anti-angiogenesis inhibitor (including bevacizumab and endostatin).

Notably, the first-line treatment group (n=12) exhibited the most favorable efficacy, with the ORR of 50% (95% CI, 25.4%-74.6%) and the mPFS of 13.3 months (95% CI, 9.0-17.6 months). In the second-line treatment group (n=6), the ORR was 16.7% (95% CI, 3.0%-56.4%), with the mPFS of 7.7 months (95% CI, 3.4-12.0 months). In the beyond the second-line treatment group (n=12), efficacy was markedly reduced with no objective responses observed (ORR = 0%, 95% CI: 0-24.2%), and the mPFS decreased to 2.6 months (95% CI, 2.3-2.9 months) (**Figures 2B**, **3**, **Table 2**). These results indicate that AK104 combination therapy achieves the greatest efficacy in the first-line setting, with PFS declining progressively as the number of prior treatment lines increases, highlighting its clinical benefit in treatment-naive patients.

### 3.3 Subgroup analysis

In the AK104 plus chemotherapy and other anti-angiogenic inhibitors group (n=9), the ORR was 33.3% (95% CI, 12.1%–64.6%), the DCR was 88.9% (95% CI, 56.5%–98.0%), and the mPFS was 11.1 months (95% CI, 0–24.4 months). Notably, the majority of patients in this group received first-line treatment (77.8%, 7/9). In comparison, patients treated with AK104 combined with chemotherapy and anlotinib (n=12) achieved an ORR of 25.0% (95% CI, 8.9%–53.2%), a DCR of 75.0% (95% CI, 46.8%–91.1%), and a mPFS of 3.0 months (95% CI, 0.3–5.7 months). Most patients in this group received second-line or later treatment (91.7%, 10/12), with a median line of therapy of 3 (range, 1–4) (**Figures 1**, **2C**, **3**, **Table 2**).

In the PD-L1-positive cohort (n=8), the ORR was 12.5% (95% CI, 2.2%-47.1%), the DCR was 87.5% (95% CI, 52.9%-97.8%), and the mPFS was 4.5 months (95% CI, 3.8-5.5 months). In the PD-L1-negative cohort (n=9), the ORR was 22.2% (95% CI, 6.3%-54.7%), the DCR was 77.8% (95% CI, 45.3%-93.7%), and the mPFS was 3 months (95% CI, 2.9-3.1 months). There was no significant difference in mPFS between the two groups (*P* = 0.76) (**Figures 2D**, **3**, **Table 2**). Among patients who had previously received PD-1 inhibitor therapy (n=17). The ORR in this subgroup was 5.9% (95% CI, 1.0%–27.0%), the DCR was 64.7% (95% CI, 41.3%–82.7%), and the mPFS was 3.0 months (95% CI, 1.5–4.8 months) (**Figures 2E**, **3**, **Table 2**).

Among patients with brain metastases (n=5), the ORR was 40.0% (95% CI, 11.8%–76.9%) and the DCR was 40.0% (95% CI, 11.8%–76.9%). The mPFS in this subgroup was 2.8 months (95% CI, 1.8–3.9 months), which was significantly shorter than that observed in patients without brain metastases, whose median PFS was 7.7 months (95% CI, 2.2–13.2 months) (*P* = 0.003) (**Figures 2G**, **3**, **Table 2**). In patients with bone metastases (n=11), the ORR was 9.1% and the DCR was 63.6% (95% CI, 35.4%-84.8%). Most patients in this subgroup received second-line or later treatment (63.6%, 7/11). The mPFS was 3.0 months (95% CI, 1.1–5.0 months), compared with 6.4 months (95% CI, 0–15.2 months) in patients without bone metastases, and this difference was statistically significant (*P* = 0.021) (**Figures 2F**, **3**, **Table 2**).

### 3.4 Safety

In this study, 25 patients (83.3%) experienced treatment-related adverse events (TRAEs) (**Table 3**), with the incidence of TRAEs varying across different combination regimens. The most common adverse events were hematologic toxicities, including anemia (66.7%), leukopenia (63.3%), neutropenia (56.7%), and thrombocytopenia (53.3%). Notably, these hematologic toxicities were consistently more frequent in the three cohorts receiving AK104 in combination with chemotherapy-containing regimens(40.0%-83.3%). In contrast, among the four patients treated with AK104 plus anlotinib alone, the incidence of hematologic TRAEs was markedly lower at 25.0%. Immune-related adverse events (irAEs), including adrenocortical insufficiency (20.0%), thyroid dysfunction (elevated TSH: 20.0% and hypothyroidism: 10.0%), and rash (13.3%), were generally low-grade and observed across all combination groups.

**Table 3:** Adverse events in the patients treated with AK104.

Adverse events	All (N = 30)		AK104+C (N = 5)		AK104+A (N = 4)		AK104+A+C (N = 12)		AK104+B+C (N = 9)	
	Any grade N(%)	Grade 3 N(%)	Any grade N(%)	Grade 3 N(%)	Any grade N(%)	Grade 3 N(%)	Any grade N(%)	Grade 3 N(%)	Any grade N(%)	Grade 3 N(%)
Any events	25(83.3)	5(16.7)	4(80.0)	2(40.0)	2(50.0)	0(0.0)	12(100.0)	2(16.7)	7(77.8)	1(11.1)
Thrombocytopenia	16(53.3)	4(13.3)	2(40.0)	1(20.0)	1(25.0)	0(0.0)	8(66.7)	2(16.7)	5(55.6)	1(11.1)
Anemia	20(66.7)	2(6.7)	3(60.0)	1(20.0)	1(25.0)	0(0.0)	9(75.0)	1(8.3)	7(77.8)	0(0.0)
Neutropenia	17(56.7)	3(10.0)	2(40.0)	1(20.0)	0(0.0)	0(0.0)	9(75.0)	1(8.3)	6(66.7)	1(11.1)
Leukopenia	19(63.3)	4(13.3)	3(60.0)	1(20.0)	0(0.0)	0(0.0)	10(83.3)	2(16.7)	6(66.7)	1(11.1)
ALT increased	7(23.3)	0(0.0)	1(20.0)	0(0.0)	0(0.0)	0(0.0)	4(33.3)	0(0.0)	2(22.2)	0(0.0)
AST increased	7(23.3)	0(0.0)	1(20.0)	0(0.0)	0(0.0)	0(0.0)	4(33.3)	0(0.0)	2(22.2)	0(0.0)
Proteinuria	4(13.3)	0(0.0)	0(0.0)	0(0.0)	1(25.0)	0(0.0)	2(16.7)	0(0.0)	1(11.1)	0(0.0)
cTnT elevation	2(6.7)	0(0.0)	0(0.0)	0(0.0)	1(25.0)	0(0.0)	0(0.0)	0(0.0)	1(11.1)	0(0.0)
Adrenocortical insufficiency	6(20.0)	1(3.3)	1(20.0)	0(0.0)	0(0.0)	0(0.0)	3(25.0)	1(8.3)	2(22.2)	0(0.0)
TSH elevation	6(20.0)	0(0.0)	1(20.0)	0(0.0)	1(25.0)	0(0.0)	2(16.7)	0(0.0)	2(22.2)	0(0.0)
Hypothyroidism	3(10.0)	0(0.0)	1(20.0)	0(0.0)	0(0.0)	0(0.0)	2(16.7)	0(0.0)	0(0.0)	0(0.0)
Hypertension	1(3.3)	0(0.0)	0(0.0)	0(0.0)	0(0.0)	0(0.0)	1(8.3)	0(0.0)	0(0.0)	0(0.0)
Rash	4(13.3)	0(0.0)	0(0.0)	0(0.0)	1(25.0)	0(0.0)	2(16.7)	0(0.0)	1(11.1)	0(0.0)
Oral mucositis	6(20.0)	0(0.0)	0(0.0)	0(0.0)	2(50.0)	0(0.0)	3(25.0)	0(0.0)	1(11.1)	0(0.0)
Diarrhea	8(26.7)	0(0.0)	1(20.0)	0(0.0)	1(25.0)	0(0.0)	4(33.3)	0(0.0)	2(22.2)	0(0.0)
Constipation	4(13.3)	0(0.0)	1(20.0)	0(0.0)	0(0.0)	0(0.0)	1(8.3)	0(0.0)	2(22.2)	0(0.0)
Leading to discontinuation	1(3.3)		0(0.0)		0(0.0)		0(0.0)		1(11.1)	
Leading to death	0(0.0)		0(0.0)		0(0.0)		0(0.0)		0(0.0)	

AK104, Cadonilimab; C, Chemotherapy; A, Anlotinib; AAI, anti-angiogenesis inhibitor (including bevacizumab and endostatin).

Grade 3 TRAEs occurred in 5 patients (16.7%), all of whom received chemotherapy-containing regimens including AK104 plus chemotherapy, AK104 plus chemotherapy and anlotinib, or AK104 plus chemotherapy and other AAI. The majority of these grade 3 events were hematologic in nature. No clear trend toward increasing grade 3 toxicity was observed across the different combination strategies. Treatment was discontinued in 1 patient (3.3%) with AK104 plus chemotherapy and anlotinib due to immune-mediated pancreatitis. Importantly, all adverse events were manageable with supportive care, and no treatment-related deaths were observed.

## 4 Discussions

Over the past decade, immunotherapy has fundamentally changed the treatment landscape of NSCLC, shifting the standard of care from later-line to first-line treatment. Durable clinical benefits have been observed across various therapeutic strategies, including immune checkpoint inhibitor monotherapy, combinations with chemotherapy, and combinations with targeted agents (8, 13). PD-1 and CTLA-4 mediate complementary but distinct immune regulation mechanisms. PD-1 blockade mainly restores the function of exhausted effector T cells within the tumor microenvironment, while CTLA-4 inhibition regulates the strength of immune responses by lowering the threshold for T-cell activation. This mechanistic complementarity provides a strong rationale for dual immune checkpoint blockade (14). Notably, multiple clinical studies have confirmed that concurrent targeting of the PD-1/PD-L1 and CTLA-4/B7 pathways significantly prolongs overall survival and improves objective response rates (10, 15, 16). As a bispecific antibody targeting both PD-1 and CTLA-4, AK104 has shown robust antitumor activity and manageable safety across several solid tumors, including cervical cancer (17), gastric cancer (9), and hepatocellular carcinoma (18). These results suggest a promising therapeutic approach that warrants further research in NSCLC.

To our knowledge, this is the first study to systematically evaluate the efficacy and safety of AK104-based combination regimens in real-world clinical practice for patients with driver gene-negative advanced NSCLC. The results demonstrated favorable clinical activity, with an ORR of 23.3%, a DCR of 80%, and a mPFS of 6.3 months. Compared to the CheckMate 9LA trial, in which nivolumab plus ipilimumab combined with chemotherapy achieved an ORR of 38% and a mPFS of 6.7 months (19), the overall ORR and PFS observed in our cohort were slightly lower. However, it is essential to note that 40% (12/30) of patients in this study had received third-line or later treatments, 16.7% (5/30) presented with brain metastases, and 94.4% (17/18) of those in the later-line subgroup had previously been treated with PD-1 inhibitors. These baseline characteristics differ substantially from those in the CheckMate 9LA trial, which predominantly included treatment-naive patients. These findings highlight that AK104-based combination therapy may provide meaningful clinical benefit for patients with driver gene-negative advanced NSCLC.

In this study, AK104 showed encouraging efficacy in combination with chemotherapy and anti-angiogenic agents. In the AK104 plus chemotherapy and other AAI treatment group (n=9), the DCR reached 88.9%, with the mPFS extending to 11.1 months. Cross-study comparisons revealed that, compared to the Phase Ib/II study of AK104 monotherapy in previously treated NSCLC (12) (ORR 0%, mPFS 1.84 months) and another retrospective study where AK104 combined with anlotinib achieved a DCR of 60% in previously treated NSCLC (20), our results with AK104 combined with chemotherapy and anlotinib (n=12) showed a DCR of 75% and mPFS of 3.0 months. Notably, the median treatment line in our study was 3 (1–4 line). As anti-angiogenic agents, VEGF inhibitors have immunomodulatory effects beyond their anti-vascular actions. They not only promote dendritic cell maturation but also improve immune cell infiltration by normalizing tumor vascular architecture, thereby reducing the immunosuppressive state of the tumor microenvironment (TME) (21–23). Triple blockade with anti-VEGF, anti-CTLA-4, and anti-PD-L1 boosts antitumor immune responses by activating B-cells and promoting the conversion of Tregs into pro-inflammatory Tregs with reduced immunosuppressive capacity (24). Chemotherapy not only reduces immunosuppressive cells but also enhances the activation and proliferation of immune cells, thereby improving the efficacy of immunotherapy (25, 26). To our knowledge, no clinical trials of such regimens have been reported in the NSCLC setting. Therefore, this study provides real-world evidence for future clinical design of combination regimens involving bispecific immune checkpoint inhibitors, chemotherapy, and anti-angiogenic agents in NSCLC.

In the first-line treatment group (n=12), the AK104 combination regimen achieved an ORR of 50% and a mPFS of 13.3 months. In contrast, several studies such as KEYNOTE-189, KEYNOTE-407, Camel, Camel-sq, and ORIENT-11 (6, 7, 27–29) reported the mPFS values of 8.0 to 11.0 months for PD-1 inhibitor plus chemotherapy. In another atezolizumab study, the combination of chemotherapy and bevacizumab achieved a mPFS of 8.3 months (30). These results indicate that the efficacy observed with AK104-based therapy was superior. We attribute this advantage to AK104 as a PD-1/CTLA-4 bispecific tumor immunotherapy agent, combined with chemotherapy and anti-angiogenic drugs in the first-line treatment regimen for most patients. Moreover, outcomes in the first-line group (ORR 50%, mPFS 13.3 months) were markedly better than in the later-line group (ORR 0%, mPFS 2.6 months). Therefore, these findings suggest that AK104 combination therapy, particularly when paired with chemotherapy and anti-angiogenic agents, offers a viable treatment option for patients with driver gene-negative advanced NSCLC.

Identifying optimal patient populations for PD-1/CTLA-4 bispecific antibodies remains a central challenge in clinical research. Existing evidence indicates that PD-L1 expression is associated with response to immune checkpoint inhibitors, but should not be used as an absolute exclusion criterion. In CheckMate 057, nivolumab significantly improved survival in patients with PD-L1-positive non-squamous non-small cell lung cancer, whereas the benefit in PD-L1-negative patients was limited (31). The POPLAR and OAK trials further demonstrated that atezolizumab conferred survival advantages across all PD-L1 subgroups, with the magnitude of benefit increasing with higher PD-L1 expression (32, 33). Notably, dual immune checkpoint blockade has expanded this paradigm. Both CheckMate 227 and CheckMate 9LA showed that nivolumab plus ipilimumab with or without limited chemotherapy improved overall survival regardless of PD-L1 status. However, the greatest survival benefit was consistently seen in patients with high PD-L1 expression, particularly those with a tumor proportion score of 50% or higher (19, 34, 35). Our findings are consistent with these observations. Among patients with driver gene-negative advanced NSCLC, the mPFS was 3.0 months in the PD-L1-negative group (TPS < 1%) and 4.5 months in the PD-L1-positive group (TPS ≥ 1%), with no significant difference between the two cohorts (*P=*0.76). This suggests that the dual-target mechanism of AK104 may partially reduce dependence on PD-L1 expression, thereby offering therapeutic potential to a broader patient population. Nevertheless, PD-L1 may still serve as a gradient indicator of treatment benefit, and future strategies should integrate additional biomarkers to enable more precise patient selection.

Previous studies have shown that tumor tissues from patients with bone metastases contain fewer immune cells, such as CD3+ T cells, CD8+ T cells, and CD68+ tumor-associated macrophages, leading to a TME that is more immunosuppressive (36). Consistent with this, subgroup analysis in this study demonstrated a significantly superior mPFS in patients without bone metastases compared to those with bone metastases (6.4 vs. 3.0 months, *P* = 0.021), suggesting that bone metastasis status is a critical factor influencing clinical efficacy. A significant difference in mPFS was also observed between patients with and without brain metastases (2.8 months [95% CI, 1.8–3.9 months] vs. 7.7 months [95% CI, 2.2–13.2 months], *P* = 0.003). This may be attributable to the limited ability of therapeutic agents to cross the blood–brain barrier. However, because the sample size of patients with brain metastases in this cohort is small (16.7%, n=5), these results should be interpreted with caution, and larger studies are required for confirmation.

In terms of safety, AK104 exhibits notable advantages attributable to its unique structural design as a PD-1/CTLA-4 bispecific antibody. Fc-engineered modifications that prevent binding to FcγRs and C1q enable the drug to avoid lymphocyte depletion and antibody-dependent cytokine release, adverse effects often linked to traditional dual immune checkpoint blockade using PD-1 and CTLA-4 monoclonal antibodies (37). This innovative mechanism was fully validated in this study. Although treatment-related adverse events (TRAEs) occurred in 83.3% (25/30) of patients, the majority were grade 1–2 hematologic toxicities, including leukopenia, neutropenia, and anemia. Notably, these toxicities were predominantly observed in regimens containing chemotherapy, whereas their incidence was substantially lower in the chemotherapy-free combination of AK104 plus anlotinib, suggesting that such events may be partially attributable to the cytotoxic chemotherapy component, rather than to AK104 alone. Only 16.7% (5/30) experienced Grade 3 events. Notably, only one patient in the entire cohort discontinued treatment due to Grade 3 immune-mediated pancreatitis, and no deaths occurred from severe adverse events. This safety profile compares favorably with the 30%-35% incidence of Grade 3–4 TRAEs reported in the dual-agent combination regimens of the CheckMate series studies (19, 35, 38). These findings confirm that the structural optimization of AK104 improves tolerability, reduces the risk of treatment discontinuation, and preserves its efficacy. This provides a more tolerable combination therapy option for patients with driver gene-negative advanced NSCLC.

This study has several limitations. First, a single-center design introduces potential selection bias and limits patient heterogeneity, while the relatively small sample size (n=30) reduces statistical power, especially in subgroup analyses such as PD-L1 expression, necessitating cautious interpretation of results. Second, retrospective data collection may lead to underreporting or recall bias in adverse event reporting. Third, the single-center design also restricts patient heterogeneity, making it hard to fully represent the varied characteristics of advanced NSCLC patients in widespread clinical practice. These limitations do not diminish the novelty of this study as the first to assess the real-world use of AK104 combination therapy in driver gene–negative NSCLC. Nevertheless, they suggest that future validation requires multicenter, prospective, large-scale randomized controlled trials. Future studies should prospectively integrate multimodal biomarker profiling including PD-L1 expression and other potential predictors to identify factors associated with durable clinical benefit and to refine patient selection for AK104-based combination regimens.

## 5 Conclusion

Despite the small sample size, this study provides preliminary evidence supporting AK104-based combination therapy as a feasible treatment option for patients with driver gene-negative advanced NSCLC. The regimen demonstrated encouraging antitumor activity, particularly in the first-line setting and when combined with chemotherapy and anti-angiogenic agents, with a favorable safety profile. These findings highlight the potential of AK104-based strategies to expand therapeutic options for this patient population and warrant further validation in larger prospective studies.

## Data Availability

The original contributions presented in the study are included in the article/supplementary material. Further inquiries can be directed to the corresponding author.
